# Lung Ultrasound B-Lines in the Evaluation of the Extent of Interstitial Lung Disease in Systemic Sclerosis

**DOI:** 10.3390/diagnostics12071696

**Published:** 2022-07-12

**Authors:** Cosimo Bruni, Lavinia Mattolini, Lorenzo Tofani, Luna Gargani, Nicholas Landini, Nicola Roma, Gemma Lepri, Martina Orlandi, Serena Guiducci, Silvia Bellando-Randone, Chiara Romei, Yukai Wang, Marco Matucci-Cerinic

**Affiliations:** 1 Department Experimental and Clinical Medicine, Division of Rheumatology, Careggi University Hospital, University of Florence, 50141 Florence, Italy; lorenzo120787@gmail.com (L.T.); lepri.gemma@gmail.com (G.L.); martinaorlandi@hotmail.it (M.O.); serenaguiducciphd@gmail.com (S.G.); s.bellandorandone@gmail.com (S.B.-R.); marco.matuccicerinic@unifi.it (M.M.-C.); 2 Department of Rheumatology, University Hospital Zurich, University of Zurich, 8006 Zürich, Switzerland; 3 Department of Experimental and Clinical Biomedical Sciences, Radiodiagnostic Unit n. 2, Careggi University Hospital, University of Florence, 50141 Florence, Italy; mattolini.lavinia@gmail.com (L.M.); nikolandini@hotmail.it (N.L.); 4 Department of Statistics, Computer Science, Applications, University of Florence, 50141 Florence, Italy; 5 Department of Surgical, Medical and Molecular Pathology and Critical Care Medicine, University of Pisa, 56126 Pisa, Italy; lunagargani@gmail.com; 6 Department of Radiology, Ca’ Foncello General Hospital, 31100 Treviso, Italy; nicola.roma@aulss2.veneto.it; 7 Department of Radiological Sciences, Oncology and Pathology, Policlinico Umberto I, Sapienza University, 00185 Rome, Italy; 8 Department of Radiology, Pisa University Hospital, 56124 Pisa, Italy; chiara.romei@gmail.com; 9 Rheumatology and Immunology Department, Shantou Central Hospital, Shantou 515031, China; stzxyywyk@126.com; 10 Unit of Immunology, Rheumatology, Allergy and Rare Diseases (UnIRAR), IRCCS San Raffaele Hospital, 20132 Milan, Italy

**Keywords:** systemic sclerosis, interstitial lung disease, computed tomography, ultrasound, radiomics

## Abstract

Background: Chest computed tomography (CT) is the gold standard for the evaluation of systemic sclerosis-related interstitial lung disease (SSc-ILD). Lung ultrasound (LUS) is a radiation-free tool that identifies the B-lines as a main feature of ILD. We aimed to investigate the role of LUS in the evaluation of the extent of SSc-ILD. Methods: Adult SSc patients underwent pulmonary function tests (PFTs), LUS and CT. The CT images were qualitatively, semi-quantitatively (the Wells score on five levels and the categorical Goh et al. staging) and quantitatively (histogram-based densitometry) analysed for ILD. LUS quantified B-lines in 21 intercostal spaces on both the anterior and posterior chest wall. Results: Out of the 77 SSc patients eligible for the study, 35 presented with ILD on CT (21 limited, 14 extensive). Total B-lines significantly differentiated ILD vs. no ILD (median 24 vs. 8, *p* < 0.001). Posterior and total B-lines significantly differentiated limited from absent ILD, while anterior B-lines distinguished extensive from limited ILD. Total B-lines correlated with the Wells score (r = 0.446, *p* < 0.001) and MLA (r = −0.571, *p* < 0.001); similar results were confirmed when anterior and posterior B-lines were analysed separately. Conclusions: LUS is a useful tool to identify SSc-ILD and to correlate with different evaluations of ILD extent and severity.

## 1. Introduction

Systemic sclerosis-related interstitial lung disease (SSc-ILD) results in significant morbidity and mortality [1,2]. Screening for the presence of SSc-ILD leads to its early identification [3], which is the first step towards the initiation of an effective treatment to slow its progression and preserve patients’ quality of life [4,5]. The development of ILD resembles other organ involvements in SSc from a pathogenetic point of view: following vascular damage and chemotactic stimuli, inflammatory cells and pro-fibrotic mediators extravasate in the perivascular tissue, consequently stimulating fibroblasts to differentiate into myo-fibroblasts and producing abnormous amounts of extra-cellular matrix components, including collagen [6].

Chest computed tomography (CT) represents the gold standard for ILD diagnosis [4], which can detect both inflammatory and fibrotic changes in the lung. Different visual methods have been proposed to quantify the extent of parenchyma affected by ILD, although these are impaired to a certain extent by inter-observer variability. Among them, the Wells score on five levels is the most frequently applied [7], particularly since it was used by Goh et al. to create a staging system classifying ILD into a limited and an extensive category, respectively [8]. In comparison to other ILD extent scores or the evaluation of the presence of certain specific ILD patterns [9], the Goh et al. staging system has a significant impact on the survival of SSc-ILD patients, with extensive ILD determining a three-times-higher risk of mortality over time, compared to limited ILD extent [8].

The use of automated software to extract data from images has progressively increased in recent decades, partially resolving the need for radiology expertise and operator variability in images interpretation [10]. Among the currently available methodologies, the histogram-based radiomic evaluation is a quantitative, operator-independent technique that relies on density distribution. The main histogram parameters that can be derived from CT images are the mean lung attenuation (MLA, representing the average global attenuation value of lung parenchyma); the skewness (SKEW, representing the degree of histogram asymmetry); and the kurtosis (KURT, indicating the degree of histogram peak). Different radiomic densitometry studies have been conducted in SSc-ILD in the last decade, showing that quantitative densitometry parameters are able to separate SSc patients with and without ILD, as well as limited versus extensive ILD according to the Goh et al. staging system [11,12]. In addition, quantitative densitometry parameters are also shown to carry prognostic implications in SSc-ILD patients, in line with mortality risk categories identified through other clinical prognostic models [13].

Given the exposure to ionizing radiation that CT entails, other techniques are currently used in clinical practice for the assessment of ILD and its follow-up. Primarily, this includes pulmonary function tests (PFTs) to detect the presence of a restrictive respiratory pattern [14] and the progression of fibrotic disease over time [15]. The use of lung ultrasound (LUS) has progressively become popular over the last decade, given its radiation-free nature and the possibility of bedside application [16]. LUS identifies the B-lines as a key feature of ILD, and they are defined as vertical and hyperechogenic lines arising from the pleural line [16]. B-lines appear as a consequence of the ultrasounds being reflected by the inter-lobular septa localized in the sub-pleural region, which are thickened as a consequence of collagen deposition. The latter alters the interface between air and lung tissue and generates the appearance of the B-lines in the LUS machine [17]. LUS has emerged as a radiation-free screening tool for SSc-ILD according to different methodological schemes of scanning sites identification and B-lines counting [18]. In addition, an LUS count of B-lines was also shown as an independent predictor of ILD onset in patients without ILD at baseline, as well as a predictor of functional worsening in SSc-ILD cases [19].

The use of LUS for the quantification of the extent of ILD has been partially explored in previous studies [20]. This was the case in the visual ILD extent scoring method of Warrick et al., with significant correlations between the increasing number of B-lines and the increasing extent/score [21,22]. Similar data were produced comparing LUS evaluation with the CT extent quantification score proposed by the Scleroderma Lung Study I and the abovementioned Wells score, although using a B-lines counting scheme with a high number of scanning sites may be time-consuming [18].

The aim of our study was to assess the role of LUS with a reduced number of scanning sites in the evaluation of the extent of SSc-ILD, compared to both CT visual scoring and CT radiomic analyses.

## 2. Materials and Methods

We retrospectively enrolled patients fulfilling the 2013 ACR/EULAR SSc classification criteria, attending the Rheumatology Unit of the Careggi University Hospital, who underwent PFTs, LUS and CT for clinical indication within 60 days. Difficulties in LUS evaluation (e.g., non-cooperating patients, a reduced lung window) or CT images un-suitable for visual or radiomic evaluation represented exclusion criteria. The local ethical committee approved the study (CEAVC 12300_oss), and patients signed informed consent. The study was conducted in agreement with the Declaration of Helsinki.

The following information was collected: demographic (age, sex, disease duration, exposure to smoking); clinical (cutaneous subset, modified Rodnan skin score, history of Raynaud’s phenomenon or digital ulcers); laboratory (positivity for the main SSc antibodies, namely anti-centromere, anti-topoisomerase I and anti-RNA polymerase III); PFT parameters (forced vital capacity, FVC%; total lung capacity, TLC%; diffusion capacity of the lung for carbon monoxide and its ratio to the alveolar volume, DLCO% and DLCO/VA%, respectively); and CT scans. The CT date constituted the reference date for data collection, with the most recent clinical evaluation being used for the demographic, clinical and laboratory data, while PFTs and LUS were eligible if performed within 60 days before or after the CT scan.

Chest CT images were eligible if acquired through a volumetric, 120 kV scan and sharp-to-very-sharp reconstruction kernel, with care dose mAs modulation and slice thickness ≤2 mm. The absence of artefacts due to movement or metal bodies represented inclusion criteria for the radiomic assessment. The images were independently assessed by two experienced thoracic radiologists (NL, NR, reviewing in consensus in case of disagreement) to verify eligibility, identify the presence of ILD (qualitative assessment) and to semi-quantify the extent of ILD (semi-quantitative assessment) using two different methods:

1. The visual Wells score at 5 levels (performed at the origin of large vessels; main carina; pulmonary venous confluence; halfway between the third and fifth section; immediately above the right hemi-diaphragm) evaluates ILD extent as an average of the 5 levels (rounded to the nearest 5% at each level) as a continuous variable ranging from 0 to 100 [7].
2. The Goh et al. staging system is based on the Wells score on CT, identifies ILD extent as a dichotomous variable (limited versus extensive) according to an average extent below or >20%. In case of indeterminate 20% clustering, FVC% <70% was used as an indicator of extensive ILD [8].

A single author (CB) performed the quantitative assessment using the free-source software Horos, as previously proposed [12]. The procedure was characterized by importing the DICOM images of the CT set into the software, setting the window view between −950 HU and −440 HU, followed by selecting the lung parenchyma in the whole images set. Finally, a dedicated plug-in was used to extract the MLA derived from each slice and to compute the total MLA, KURT and SKEW from the whole set of images.

**Figure 1:**
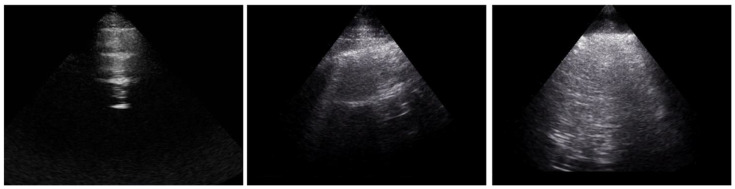
Example of normal LUS (**left panel**), LUS with 1 B-line (**central panel**) and with multiple B-lines (**right panel**).

The B-lines were separately quantified with LUS by two assessors (LG, GL), who examined 21 intercostal spaces from the posterior (13 spaces) and anterior (8 spaces) chest wall. Examples of the assessment of LUS space are presented in Figure 1. The sum of anterior and posterior B-lines determined the total B-lines number.

All assessors were blinded to the results of the other two evaluations.

Continuous variables were presented using mean ± standard deviation or median (interquartile range), categorical variables using absolute and relative frequencies. To evaluate the difference between continuous variables among two groups, the T-test, Satterthwait’s test or Mann–Whitney test was used, according to the result of the Shapiro–Wilk test for normality and the F-test for the equality of variances. To assess the difference between continuous variables among three groups, ANOVA, Welch ANOVA or the Kruskal–Wallis test was used according to the result of the Shapiro–Wilk test for normality and the Bartlett’s test for homoschedasticity. Multiple comparisons between groups were performed using Tukey, Games–Howell or Dwass–Steel–Critchlow–Fligner tests, respectively. We used Pearson’s correlation coefficient to evaluate the correlation between two continuous variables. The area under the receiver operating characteristics curve (AUC) was used to assess the predictive value of the independent variable (FVC, DLco, B-lines) for the outcome (presence of extensive ILD). The statistical significance level was set to 5%.

## 3. Results

Out of the 94 SSc patients enrolled, 13 were excluded as CT images were not available at the time of the visual scoring evaluation, while 4 had limitations in LUS performance. The resulting study population included 77 subjects (65 females, age 48 ± 16 years; see Table 1 for further clinical characterization).

Signs of ILD on qualitative visual examination were detected in 35 (46%) patients, and the Wells score ranged from 0 to 65% of total ILD extent in the whole group, with a median value in the ILD population of 10 (5–30%). ILD extent was categorized as limited extent in 21/35 (60%) cases and extensive in 14/35 (40%), according to the Goh et al. staging system. When analysed with the Horos software, the CT images of 18 patients were excluded from the analysis due to the presence of movement artefacts/metal bodies, which may lead to the misrecognition of extra-pulmonary tissues as lung parenchyma. This led to the radiomic analysis being restricted to 59 cases (77% of the study population).

In patients with SSc-ILD, significantly lower PFT parameters were detected when compared to patients without ILD: FVC% (93 ± 28 vs. 105 ± 18, *p* = 0.006); TLC% (86 ± 17 vs. 101 ± 15, *p* = 0.021); DLCO% (60 ± 21 vs. 83 ± 16, *p* < 0.001); and DLCO/VA% (72 ± 17 vs. 84 ± 17, *p* < 0.001), as expected. Similarly, the number of anterior [14 (7; 22) vs. 4 (1; 6), *p* < 0.001], posterior [12 (5; 24) vs. 2 (0; 9), *p* < 0.001] and total [24 (13; 46) vs. 8 (1; 15), *p* < 0.001] B-lines significantly differentiated ILD vs. non-ILD patients. Similarly, the radiomic parameters distinguished ILD versus non ILD-patients: this was statistically significant for MLA [−807 (−776; −820) HU vs. −836 (−844; −820) HU; *p* < 0.001]; KUR [7.6 (2.5; 15.6) vs. 14.6 (9.0; 22.7), *p* = 0.027]; and SKEW [ 2.5 (1.5; 3.4) vs. 3.4 (2.7; 4.2), *p* = 0.028].

When ILD patients were clustered according to the Goh et al. staging system, PFT parameters, in particular FVC% and TLC%, were different among the groups and significantly differentiated extensive from absent ILD. In addition, both DLCO% and DLCO/VA% significantly differentiated the limited from the extensive ILD groups, but not between the absence of ILD and limited ILD extent and absence of ILD groups (Table 2).

**Figure 2:**
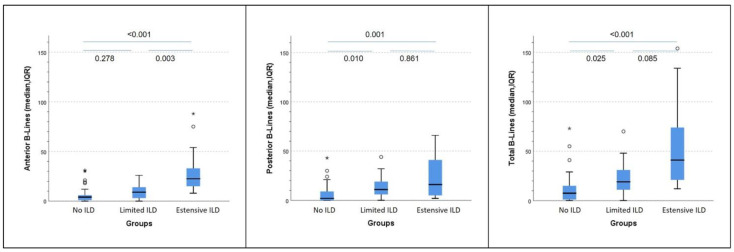
Distribution of anterior, posterior and total B-lines among the patients with absent, limited and extensive interstitial lung disease. ILD = interstitial lung disease. Statistical significance is set for *p* < 0.05. ° represent mild outliers, while * represent extreme outliers, in relation the distribution of B lines within the group.

Conversely, the number of posterior and total B-lines significantly distinguished limited from absent ILD, while the number of anterior B-lines differentiated extensive from limited ILD. A trend towards statistical significance (*p* = 0.085) was also seen for the total number of B-lines in distinguishing between extensive and limited ILD (Figure 2).

**Figure 3:**
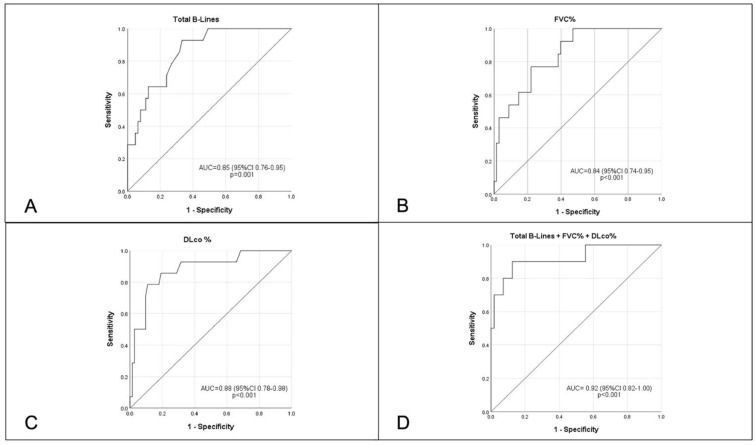
Receiver Operating Characteristics curves for the prediction of presence of extensive interstitial lung disease on high-resolution computed tomography, including Total B-lines (Panel **A**), FVC% (Panel **B**), DLco% (Panel **C**) and the combination of FVC%, DLco% and Total B-lines (Panel **D**). DLco% = diffusion lung capacity of carbon monoxide; FVC% = forced vital capacity.

When tested separately, the number of total B-lines (AUC 0.85, 95% CI 0.76–0.95, *p* < 0.001, Figure 3A), FVC% (AUC 0.84, 95% CI 0.74–0.95, *p* < 0.001, Figure 3B) and DLCO (AUC 0.88, 95% CI 0.78–0.98, *p* < 0.001, Figure 3C) significantly predicted the presence of extensive ILD on HRCT, with a further increase in the AUC when the three tests were combined at the same time (AUC 0.92, 95% CI 0.82–1.00, *p* < 0.001, Figure 3D). Although numerically superior, no statistically significant difference was seen between using the single test or the combination of the three assessments to predict the presence of extensive ILD, given the small sample size.

In line with the visual scoring evaluation, MLA significantly differentiated the three clusters of ILD cases (extensive, limited and absent), while KUR and SKEW only differentiated extensive from absent ILD.

Weak-to-moderate correlations were found between the three B-lines assessments (anterior, posterior and total) and all PFT parameters, the Wells visual scoring and the three radiomic parameters. In detail, there was a statistically significant negative correlation between anterior, posterior and total B-lines with all PFT parameters, SKEW and KUR, while there was a positive statistically significant correlation with MLA and the Wells Score (Table 3).

## 4. Discussion

Our study confirms the usefulness of LUS in differentiating between the presence and absence of ILD and further supports its role in ILD extent assessment compared to CT semi-quantitative and radiomic quantitative evaluations.

**Figure 4:**
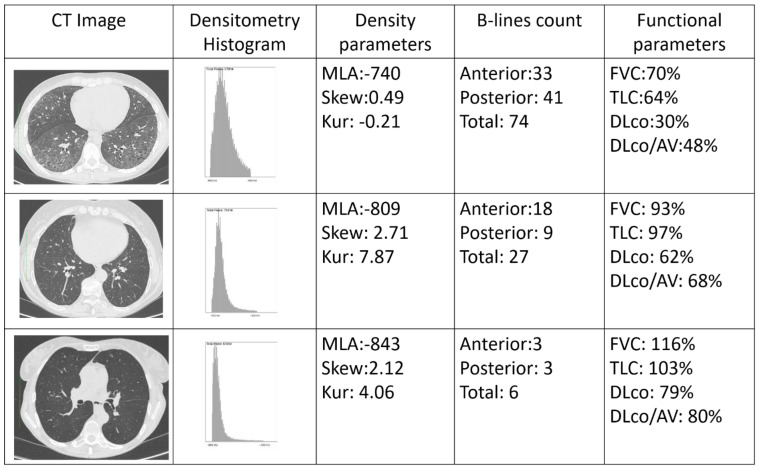
Example of systemic sclerosis patients with extensive (**first-line**), limited (**second-line**) and absent (**third-line**) interstitial lung disease. CT = computed tomography; DLco = diffusion lung capacity of carbon monoxide; DLco/VA = diffusion lung capacity of carbon monoxide corrected for alveolar volume; FVC = forced vital capacity; KUR = kurtosis; MLA = mean lung attenuation, measured in Hounsfield unit; SKEW = skewness; TLC = total lung capacity.

LUS is a radiation-free, non-invasive, bedside technique which has been studied in different conditions, including SSc. The number of B-lines distinguished between the presence and absence of SSc-ILD in previous publications, including early sub-clinical cases [23,24]. In addition, some authors have shown the relationship between the quantification of B-lines and the visual CT ILD extent scoring method by Warrick et al. using the Scleroderma Lung Study I scoring system, with higher numbers of B-lines in patients with higher ILD extent [18,21,22,25]. In comparison to previous reports, we confirm the correlation with the CT Wells score and the number of ultrasound B-lines, although with lower coefficients, possibly related to the use of a different number of scanning sites. In comparison to the other CT visual quantitative scoring methods, the classification into limited or extensive ILD according to the Goh et al. staging system carries prognostic implications [8]. In our population, the number of anterior B-lines distinguished limited and extensive ILD, with a trend towards statistical significance for the total B-lines count. Similarly, posterior and total B-lines significantly differentiated the presence of limited ILD from the complete absence of ILD. These results may be related to the natural history of SSc-ILD, which initially affects posterior-basal peripheral areas and progressively expands to anterior and proximal segments of the lung parenchyma, further supporting the ability of LUS to also detect early sub-clinical interstitial involvement and indicating its role in detecting a progression of ILD extent. In line with previous reports, PFT parameters (in particular DLCO% and DLCO/VA%) significantly distinguished extensive from limited ILD, although they did not differ between limited and absent ILD. Conversely, FVC% and TLC% can significantly separate extensive versus absent ILD only, confirming that they may be associated with a delay in detecting milder forms of ILD [26]. Therefore, B-lines may add information to the sole use of PFTs, in particular to support the differentiation between absent, limited and extensive ILD, providing anatomical details in a functional evaluation (Figure 4).

The role of B-lines in evaluating the extent of SSc-ILD is corroborated by the correlations with the CT radiomic quantitative indexes. Ariani et al. identified significantly higher values of MLA and lower values of both SKEW and KUR in SSc-ILD patients, compared to SSc cases without lung fibrosis [12]. Our data not only confirms the previously shown ability of MLA to distinguish extensive from limited ILD [11,12], but also shows the ability of the radiomic assessment to distinguish limited versus absent ILD. As for the Wells score, a statistically significant correlation was also found between the three radiomic parameters and the three B-lines counts, although this was moderate for MLA and weak for both SKEW and KUR. In comparison to Ariani et al., our radiomic analysis included all the images in each CT set. Although this resulted in weak correlations with other methods focusing on specific lung areas (see the LUS scanning scheme, the Wells score and the Goh et al. staging system), our radiomic analysis obtained values that were more representative of the whole lung parenchyma and not only standardized levels.

Our study has some limitations: the small sample of patients did not allow for the creation of prediction models combining PFTs and LUS. In addition, LUS can only evaluate the sub-pleural regions of the lung, thus potentially explaining the weak-to-moderate correlations we found with both the CT Wells score, semi-quantitative assessment and the radiomic assessment, which both also evaluate central lung portions. In addition, CTs were performed with different scanners and protocols, potentially biasing both the radiomic and visual analyses. Finally, the LUS assessment was performed by a single operator, although it has been shown that this methodology carries very high values of both inter- and intra-reader reliability [16,24].

In conclusion, our preliminary study proposes the synergic use of PFTs and LUS in the assessment of SSc-ILD extent. This should be investigated further in prospective studies on larger populations to support its application in clinical practice and reduce the radiological burden of SSc-ILD assessment.

## Figures and Tables

**Table 1:** Characteristics of the study population.

Parameters	Distribution among the Whole Cohort (*n* = 77)
Age, years, mean ± SD	48 ± 16
Disease duration, years, median (IQR)	4 (1; 6)
Female sex, n (%)	65 (84)
Diffuse cutaneous subset, n (%)	10 (13)
Anti-centromere antibody positive, n (%)	32 (42)
Anti-topoisomerase I antibody positive, n (%)	29 (38)
Anti-RNA polymerase III antibody positive, n (%)	1 (1)
Digital ulcers (ever), n (%)	29 (38)
Raynaud’s phenomenon, n (%)	69 (90)
NYHA functional class ≥2, n (%)	31 (43)
Smoking exposure (ever), n (%)	35 (46)
Interstitial lung disease on CT, n (%)	35 (46)
Extensive ILD on CT according to Goh et al. [8], n (%)	14 (18)
Wells score on 5 levels [7], median (IQR)	10 (5; 30)

CT = chest computed tomography; ILD = interstitial lung disease; IQR = interquartile range; NYHA = New York Heart Association; SD = standard deviation.

**Table 2:** Distribution of pulmonary functional parameters, B-lines and radiomic parameters among the study population and stratified according to lung involvement. Patient numbers in brackets refer to the subgroup with available radiomic data.

Parameters	Distribution among the Whole Cohort; *n* = 77 (59)	No ILD *n* = 42 (34)	Limited ILD *n* = 21 (16)	Extensive ILD *n* = 14 (9)	
FVC%, mean ± SD	100 ± 18	105 ± 18	98 ± 15	85 ± 16	§ *
TLC%, mean ± SD	98 ± 17	101 ± 15	98 ± 15	82 ± 17	§ *
DLCO%, mean ± SD	76 ± 21	83 ± 16	76 ± 20	51 ± 16	§ ^ *
DLCO/VA%, mean ± SD	81 ± 18	84 ± 17	83 ± 18	67 ± 14	§ ^ *
Anterior B-lines, median (IQR)	6 (2–16)	4 (1–6)	9 (2–16)	23 (15–38)	§ ^ *
Posterior B-lines, median (IQR)	7 (0; 15)	2 (0; 9)	11 (5; 20)	16 (5; 42)	§ * °
Total B-lines, median (IQR)	14 (5; 29)	8 (1; 15)	19 (11; 32)	41 (21; 80)	§ * °
MLA, HU, median (IQR)	−823 (−838; −807)	−836 (−844; −820)	−820 (−829; −801)	−780 (−761; −810)	§ ^ * °
SKEW, median (IQR)	2.8 (2.1; 4.0)	3.4 (2.7; 4.2)	2.6 (2.2; 3.5)	1.6 (0.4; 2.4)	§ *
KUR, median (IQR)	10.9 (4.6; 20.9)	14.6 (9.0; 22.7)	7.8 (5.9; 16.2)	3.7 (−0.62; 8.9)	§ *

§ significant difference between three groups, with *p* value < 0.05; ^ significant difference between extensive and limited ILD groups, with *p* value < 0.05; * significant difference between extensive and no-ILD groups, with *p* value < 0.05; ° significant difference between limited and no-ILD groups, with *p* value < 0.05; DLCO = diffusion lung capacity of carbon monoxide; DLCO/VA = diffusion lung capacity of carbon monoxide corrected for alveolar volume; FVC = forced vital capacity; HU = Hounsfield unit; ILD = interstitial lung disease; IQR = interquartile range; KUR = kurtosis; MLA = mean lung attenuation; SKEW = skewness; TLC = total lung capacity.

**Table 3:** Correlations between B-lines and pulmonary function tests, densitometry parameters and the visual score.

Parameters	Anterior B-Lines	Posterior B-Lines	Total B-Lines
FVC%	r = −0.383 *p* = 0.005	r = −0.420 *p* = 0.004	r = −0.468 *p* < 0.001
TLC%	r = −0.401 *p* = 0.006	r = −0.502 *p* < 0.001	r = −0.435 *p* < 0.001
DLCO%	r = −0.483 *p* < 0.001	r = −0.483 *p* < 0.001	r = −0.511 *p* < 0.001
DLCO/VA%	r = −0.341 *p* = 0.010	r = −0.286 *p* = 0.047	r = −0.303 *p* = 0.024
MLA	r = 0.519 *p* < 0.001	r = 0.559 *p* < 0.001	r = 0.568 *p* < 0.001
SKEW	r = −0.311 *p* = 0.015	r = −0.386 *p* = 0.001	r = −0.368 *p* = 0.004
KUR	r = −0.252 *p* = 0.050	r = −0.285 *p* = 0.027	r = −0.283 *p* = 0.028
Wells score on five levels	r = 0.443 *p* < 0.001	r = 0.386 *p* < 0.001	r = 0.436 *p* < 0.001

Statistical significance is set for *p* < 0.05. DLCO = diffusion lung capacity of carbon monoxide; DLCO/VA = diffusion lung capacity of carbon monoxide corrected for alveolar volume; FVC = forced vital capacity; KUR = kurtosis; MLA = mean lung attenuation; SKEW = skewness; TLC = total lung capacity.

## Data Availability

Data can be made available upon reasonable request.
